# Two cases of low birth weight infant survival by prehospital emergency hysterotomy

**DOI:** 10.1186/s13049-017-0407-8

**Published:** 2017-07-03

**Authors:** Miretta Tommila, Mikko Pystynen, Hanna Soukka, Fatih Aydin, Matias Rantanen

**Affiliations:** 1 Emergency Medical Services, FinnHEMS 20, University of Turku and Turku University Hospital, Savitehtaankatu 1, Turku, Finland; 2 Emergency Medical Services, Department of Emergency Care, University of Helsinki and Helsinki University Hospital, Helsinki, Finland; 3 Division of Perioperative Services, Intensive Care Medicine and Pain Management and Department of Anaesthesiology and Intensive Care, University of Turku and Turku University Hospital, Turku, Finland; 4 Department of Pediatric and Adolescent Medicine, Turku University Hospital, Turku, Finland

**Keywords:** Perimortem caesarean delivery, Resuscitative hysterotomy, Prehospital emergency hysterotomy, Maternal cardiac arrest

## Abstract

**Background:**

During maternal cardiac arrest, emergency hysterotomy (EH) is recommended after four minutes of resuscitation, if no signs of spontaneous circulation are detected. This extreme procedure is believed to be potentially beneficial for both the mother and the infant. Both maternal and neonatal survivals seem to be associated to the time delay between the cardiac arrest and the delivery and in-hospital resuscitation location. In addition to this, gestational age is an important determinant to neonatal outcome.

**Case presentation:**

We report two emergency hysterotomies executed in an out-of-hospital location. The infants delivered by EH were low birth weight infants and born 20–23 min after maternal cardiac arrest. Both infants survived and had normal physical and neurological growth at the age of two years. Unfortunately, mothers in these both cases died in the field.

**Conclusion:**

Contrary to earlier beliefs, it is possible to perform a successful EH also in out-of-hospital setting, even with incomplete surgical skills. However, training and preparation are extremely important for achieving the highest possible readiness to treat maternal cardiac arrest situations also prehospitally.

## Background

Cardiac arrest during pregnancy is a rare, but extremely dramatic occasion. Causes may be obstetric, non-obstetric, traumatic, or even iatrogenic [1, 2]. Advanced life support principles in pregnant women are similar to those in non-pregnant population, but in addition to other resuscitative actions, European Resuscitation Council guidelines [3] recommend to perform a caesarean section after 4 min regardless of the cause of cardiac arrest, if initial resuscitation attempts seem to fail. According to the recommendations, the infant should be delivered within one minute.

There are many factors, such as a time delay to reach the patient, challenging working environment, and lack of skills and equipment, which make emergency hysterotomy out-of-hospital even more difficult than in-hospital. Despite these difficulties, successful EH is possible also prehospitally. Here we describe two cases of prehospital emergency hysterotomies during maternal cardiopulmonary resuscitation with a lamentable loss of both mothers, but an extraordinary survival of low birth weight infants in both cases.

## Case presentation

### Case 1

In December 2014, 38-year-old female (gravida 2 para 1) had suddenly fallen to the ground on the street. Couple of people witnessed the incident. One of them called to the emergency response centre. The dispatcher instructed to check if the patient reacts and breaths normally while she alerted an ambulance. After finding out there was no signs of life, the dispatcher gave instructions for basic life support, and alerted also the EMS supervisor unit, and the HEMS unit according to the predetermined protocol, and in addition to that, one more ambulance, which happened to be free and driving near the scene, for extra help. The EMS supervisor was accompanied by an emergency medicine resident and the additional ambulance by an anaesthesiology and intensive care resident, both residents were on prehospital training rotation. Thus the prehospital team was exceptionally abundant.

The first ambulance arrived to the scene within 2 min from the emergency call. At that time, the bystanders did compressions, but not ventilation. The first paramedics applied cardiac monitoring and started advanced life support according to the Finnish guidelines, which are based on the European Resuscitation Council Guidelines [4]. Because the initial rhythm was asystole, cardiopulmonary resuscitation was performed according to the non-shockable rhythm protocol.

During the resuscitation, the state of pregnancy was noticed. According to the maternity card, the gestational weeks of the patient were 30 + 4. After this observation, the uterus was tilted to the left manually to reduce the aortocaval compression.

The second ambulance and the EMS supervisor unit arrived to the scene a couple of minutes later. The cardiopulmonary resuscitation was continued. During the resuscitation, the patient was intubated and manually ventilated at 1,0 inspired oxygen (FiO_2_). Intravenous access was opened and adrenaline was given as 1 mg intravenous boluses according to the protocol at total of five times. After the first cycle, the rhythm was pulseless electrical activity diagnosed by cardiac monitoring and palpating the carotid pulse. Despite the advanced life support, the patient showed no signs of life.

The HEMS doctor was informed about the situation before arrival to the scene. At that point, ALS had continued for 15 min. Because of the lack of experience the HEMS doctor called the obstetrician on duty, and in agreement they decided that emergency hysterotomy should be performed. The HEMS doctor had no previous experience from caesarean sections, so the obstetrician stayed on line and gave instructions via a cellphone. There was no predetermined protocol for EH, so the cellphone consultation was a coincidental idea.

The HEMS unit arrived to the scene 18 min after the cardiac arrest, and operation preparations were started immediately. During the preparations, we performed quick ultrasound (SonoSite Nanomaxx) examination for 5 s in order to be sure that fetus was alive (because it has been 18 min of CPR) and fetal heart beats were established. The ALS of the mother was continued efficiently under the direction of the EMS supervisor.

At 21 min after the cardiac arrest, the abdomen was opened with a relatively large vertical midline incision according to the instructions of the obstetrician via the cellphone. The peritoneal layer was opened bluntly. The uterus was cut with a lengthwise incision, and a female infant was extracted at 23 min after the cardiac arrest. There were no signs of bleeding at any point of the operation.

Right after the birth, the infant was hypotonic, apneic, and cyanotic. The infant was moved to the preheated ambulance for resuscitation. She was wiped and the airways were suctioned. A low heart rate less than 60/min was observed. Bag-mask ventilation was started first with room air, then with 100% oxygen. Chest compressions were started within the first minute. Because of no response to resuscitation efforts, orotracheal intubation with a 2.5 tube was performed and intravenous access was opened. Ringer’s acetate 30 ml was given because of possible hypovolaemia.

After a few minutes the colour of the infant started to improve and she started to gasp. Twelve minutes after the delivery, ROSC was achieved. The positive pressure ventilation was continued and the infant was kept as warm as possible.

ALS of the mother was continued after the delivery by the EMS supervisor and his team, but there were no signs of improvement. Pulmonary embolism was speculated as a possible cause for the sudden cardiac arrest, but because of the long resuscitation time and poor clinical prognosis no more interventions were done, and ALS was ceased after 35 min of the cardiac arrest. Afterwards it was found out that the mother had a previous history of congenital heart disease operation, and later autopsy findings revealed a rupture of the aorta as the cause of death.

The infant was transferred to the neonatal intensive care unit of the University Hospital. On admission, her weight was 1400 g, capillary blood pH 7.16, pCO_2_ 4.9 kPa and rectal temperature 32.9 °C. The blood glucose was 4.7 mmol/l. She was connected to a ventilator and one dose of surfactant was administered. She was started on restricted fluids and antibiotics. Her clinical course was uneventful during the whole hospital stay. She was extubated during her first day of life, started to urinate during the first 24 h and was on full enteral feeds on day of life 10. She had no infection-related laboratory findings and the antibiotic treatment was discontinued after two days. Repeated brain ultrasound examinations and neurologic examinations were normal. Furthermore, her brain MRI performed at term-equivalent age was normal. She was discharged from the hospital at the age of 7 weeks, and at the age of two years, her neurological development and physical growth were normal.

### Case 2

A 31-year-old healthy female suddenly collapsed with short lasting convulsions in January 2015. Patient’s second pregnancy was at gestational weeks 26 + 5. Her first pregnancy had been uneventful and had ended in normal childbirth. At the time of collapse patient was at home with her spouse who immediately called emergency response center. Dispatcher alerted nearest ambulance capable for basic life support within 60 s from beginning of the emergency call. A cardiac arrest was not identified during the emergency call, but after six minutes from beginning of the call the dispatcher alerted the mobile intensive care unit (MICU) according to the alert protocol of unconscious patient. MICU is normally staffed by two paramedics and an emergency physician. However, there was also an extra paramedic in MICU due to rotation of ambulance staff. The emergency physician on duty was a resident of anesthesiology and intensive care.

The first ambulance reached the patient 10 min from beginning of the emergency call. Patient was lying on a couch unconscious and not breathing. No CPR was performed by bystander. Patient was moved to the floor by the EMS personnel. The primary rhythm was ventricular fibrillation and cardiopulmonary resuscitation was carried out according to the shockable rhythm protocol. Before arriving to the scene, the MICU emergency physician was informed about the cardiac arrest of a pregnant patient. A plan of first establishing advanced life support and then deciding on the need for prehospital emergency hysterotomy was made. Emergency physician on duty had no previous experience from caesarean sections.

MICU reached the patient 15 min from beginning of the emergency call. VF had been defibrillated two times and second two minute cycle of compression was going on. A left lateral tilt of uterus was performed. Patient was intubated and intravenous line was established by the MICU staff. Because of long delay of ALS and no bystander CPR fetal heart beats were checked with an ultrasound device (SonoSite M-Turbo) and were found to be approximately 60 times per minute. The examination was performed during establishing of an intravenous line and report of first responder unit and caused no delay for EH. The decision of prehospital EH was made by the emergency physician and the procedure was performed during ALS of the mother. 18 min from beginning of the emergency call a horizontal abdominal skin incision was made followed by blunt approach to uterus. Uterus was then opened with small horizontal incision extended with fingers. A female infant was born two minutes after the skin incision. There was no bleeding during the operation.

During the EH an additional ambulance and the EMS supervisor were alerted by request of the emergency physician. After the arrival of the additional units a total of nine paramedics and emergency physician were on scene. ALS of the mother continued also during the operation. Amiodarone was prepared to be administered but not given due to asystole following the third defibrillation. Adrenaline was given at 1 mg doses according to the protocol in a total of 5 times. Rhythm was mainly a pulseless electrical activity with no ventricular contractions detected by ultrasound. There was no return of spontaneous circulation and resuscitation was stopped at 49 min from the beginning of the emergency call. Cause of the ventricular fibrillation remains unclear even after autopsy.

The infant was cyanotic and not breathing. Low pulse rate was still palpable from the umbilical artery. Airways were suctioned and mask ventilation started. Chest compressions were also started after one minute because the rate of pulse was still low. During the resuscitation, an orotracheal intubation was performed and intraosseous needle was set. After two minutes of CPR the pulse rate increased to 120 per minute and spontaneous breathing efforts and body movements were noticed.

The infant was transported to the neonatal intensive care unit of the University Hospital. Despite the hard efforts to maintain body temperature, the temperature was only 30 °C at time of arrival to intensive care unit. Her birthweight was 950 g. On admission, the arterial blood gas analysis showed combined metabolic and respiratory acidosis. pH was 7.05, base excess −14 mmol/l, CO_2_ 8.5 kPa, O_2_ 7.5 kPa and lactate 8.8 mmol/l. Blood glucose was 5.3 mmol/l. The metabolic acidosis was successfully corrected within 12 h after admission. Due to low thromboplastin time, also frozen plasma was administered. Pulmonary gas exchange remained impaired and respiratory distress syndrome (RDS) was diagnosed. Surfactant was administered twice. The pulmonary status improved within the first week of life and the infant was extubated at the sixth day after admission.

After weaning from the invasive ventilation intermittent nasal CPAP was applied. Repeated brain ultrasound remained normal until age of five days when a bilateral grade II intraventricular hemorrhage was diagnosed. However, the hemorrhage was almost completely resorbed before discharge from the intensive care unit to a neonatal ward at the age of four weeks. There were no signs of infection. She was discharged from the hospital at the age of two and half months. Brain MRI at the age of four months was normal. At the age of two years she was healthy and her neurological development was normal for her age.

## Discussion and conclusions

After 20 weeks of gestation, the growing uterus can compress inferior vena cava and aorta, thus impending venous return and cardiac output. For this reason, left uterine displacement is recommended during CPR [5] The efficacy of this maneuver in cardiac arrest situations is unclear, and therefore an early evacuation of a fetus is considered to be beneficial for the mother after 20 weeks, and for both the mother and the infant after 24–25 weeks of gestational age [3].

The science behind maternal resuscitation guidelines is understandably sparse, and based mainly on reviews, case reports and simulation based studies [3, 6–8]. The most important factors affecting maternal survival seem to be in-hospital location and emergency hysterotomy in less than 10 min [9]. Neonatal survival is associated with increasing gestational age [2], but also the time interval from cardiac arrest to delivery seems to be critical [9]. However, in few case reports, exceptionally long time intervals, up to 30–45 min from cardiac arrest to emergency hysterotomy, have been described with normal neurological outcome at the age of 4 years (30 min from CA) and 6 months (45 min from CA) [10, 11].

In addition to time related matters, the setting of the maternal cardiac arrest is also an important factor affecting neonatal survival. In-hospital neonatal survival has been reported to be significantly higher than out-of-hospital survival [9]. Thus, a few case reports of prehospital emergency hysterotomies have been published [12–14]. In these out-of-hospital cases, only one infant has made a secondary survival with a slightly reduced neurological development at the age of four years, one infant died on the scene and one after first 24 h.

An absolute time frame after which not to attempt an emergency hysterotomy is difficult to define. The four-minute rule of EH has recently been challenged [15]. Efficient maternal CPR can produce only 10% of the normal cardiac output [2], but a fetus has multiple physiologic mechanisms to respond to hypoxic sequelae lasting more than 10 min, including high hemoglobin concentration, increased oxygen affinity, and the ability to direct the blood supply to the brain, heart, and adrenal glands when needed [1]. This is remarkable especially in a prehospital setting, where the time delay to reach a patient in a maternal CA might often be longer than 4–5 min.

EH has been considered to be potentially beneficial to the mother, but never harmful [9]. The most common neonatal outcomes after EH are either surviving intact, or not surviving at all [16]. These findings might diminish the pressure of critical decision making. Without procedures, both a mother and an infant will probably be lost. By performing an EH, lives might be saved, and a risk for severe neurological impairment seems to be minor.

Although higher gestational age has been associated to a better neonatal survival, the infants of the both cases were low birth weight infants. At a higher gestational age, infants will be more mature for extrauterine life. At an earlier gestational age, problems related to prematurity, like respiratory distress syndrome and temperature loss, are more likely to appear. On the other hand, earlier in pregnancy the uterus is probably smaller, causing perhaps less deterioration to venous return and cardiac output, and allowing more effective impact of maternal CPR efforts.

With our two cases, we wish to encourage other physician-staffed EMS units to consider the possibility of EH in maternal cardiac arrest situations. In these cases, the procedure itself was successful without earlier surgical experience. However, these cases are not descriptions of an ideal performance of EH. At least a few meritious papers [2, 8, 17] have reviewed earlier literature and published recommendations for how to perform an EH.

The most of all, we want to emphasize the importance of training and preparation for EH, like for any other rare and critical situations. Without training or predetermined protocols any management is in a great risk to be hesitant, and depends on thoughts and actions of an individual. For example, the first EH was performed with a vertical incision, when a horizontal incision was used for the second EH. Both incision strategies lead to a similar result, but the literature suggests a vertical midline laparotomy incision might be better for emergency situations [2, 8, 17] in order to maximize exposure and make the procedure as easy as possible. A beforehand preparation could make procedure more structured and diminish variation between different operators. An important aspect is also relieving a mental workload of an operator, because often the most difficult thing in a critical situation is to make the decision. Simulation training, beforehand preparation [18], and mental imagery rehearsal are the keys to achieve a readiness to perform rare procedures [2] also in out-of-hospital locations.

## Abbreviations

ALS: Advanced life support
CA: Cardiac arrest
CPR: Cardiopulmonary resuscitation
EH: Emergency hysterotomy
EMS: Emergency medical services
HEMS: Helicopter emergency medical services
MICU: Mobile intensive care unit
